# Simulation and Experiment on the Low-Velocity Impact Response of Flax Fabric Reinforced Composites

**DOI:** 10.3390/ma16093489

**Published:** 2023-04-30

**Authors:** Xiaoshuang Xiong, Zisheng Wang, Zihao Zhang, Qiaomin Li, Chen Shen, Fei Fan, Xiang Li, Mingzhang Chen

**Affiliations:** 1 Hubei Key Laboratory of Digital Textile Equipment, Wuhan Textile University, Wuhan 430200, China; xsxiong@wtu.edu.cn (X.X.); qmli@wtu.edu.cn (Q.L.);; 2 School of Mechanical Engineering & Automation, Wuhan Textile University, Wuhan 430200, China; 3 China Special Equipment Inspection and Research Institute, Beijing 100029, China; 4 Hubei Key Laboratory of Advanced Technology for Automotive Components, Wuhan University of Technology, Wuhan 430070, China

**Keywords:** flax fabric reinforced composites, low-velocity impact, multi-scale, numerical simulation

## Abstract

Natural fiber reinforced composites are increasingly used to fabricate structural components prone to suffering low-velocity impacts. The low-velocity impact response of flax fabric reinforced composites under different impact energies is experimentally studied and numerically simulated. A multi-scale finite element analysis strategy for the progressive damage prediction of flax fabric reinforced composites is developed. Micro- and meso-scale analyses are conducted to predict the effective properties of the woven unit cell. Macro-scale analysis is carried out subsequently to predict the impact response of composite laminates using the results of micro- and meso-scale analyses as inputs. Simulation results and experimental results both show that most of the impact energy is absorbed by the specimens when the impact energy is lower than 4 J, and the absorption ratio of impact energy slightly increases with the increase in impact energy. On the contrary, a dramatic decrease occurs in the absorption ratio when the impact energy is 6 J, due to the severe damage to the specimen. In addition, simulation results indicate that matrix shear damage and interlaminar damage are the primary failure modes of composites under high impact energy. The numerical results of impact force, absorbed energy, and damage morphologies on both sides for all specimens show good agreement with the experimental results.

## 1. Introduction

Over the last decade, natural fibers have been increasingly used as reinforcement in polymer composites because of their promising properties, such as high specific stiffness, good acoustic insulation, low cost, and less environmental impact [1,2]. Some natural fibers, such as flax and hemp, have been considered viable alternatives to glass fibers because of their good mechanical properties and lower cost [3]. Natural fiber reinforced composites are used in automobile components such as door panels, seat backs, dashboards, pillar covers, engine insulation, headliner panels, and parcel shelves [4,5,6,7,8]. Recently, the potential of natural fiber fabric reinforced polymer composite laminates in structural applications has been increasingly explored by researchers and engineers, for example, as wind turbine blades [9], automobile hoods [10], and aeronautic cowlings [11].

Generally, composite laminates applied to structural components are prone to suffering low-velocity impacts such as tool drops, stone strikes, or debris strikes, and then damage occurs in the materials. The type and size of damage caused by an impact usually determine the residual properties of the structure made of composite laminates [12,13,14,15]. Therefore, the low-velocity impact behavior of composite laminates has always been an important design consideration in many applications. For the complex architectures of the textile fabrics in the natural fabric reinforced composites, many factors will influence the low-velocity impact performance of the materials, for example, material properties of the fiber, yarn, and matrix, weaving/braiding construction, fiber packing density in the yarn, and overall fiber volume fraction [15].

Recently, numerous studies have been carried out to investigate the failure characteristics of natural fiber reinforced composites under impact loading [15,16,17,18,19]. Liang et al. [19] investigated the low-velocity impact behaviour and residual properties of quasi-isotropic flax/epoxy composites. They concluded that the fracture mechanism consisted of the delamination that occurred at a low energy level, followed by the development of intra-laminar transverse cracks with the increase of impact energy, and a maximum loss of 15–30% in compression resistance was noticed for a high impact energy of 10 J. Habibi et al. [20] further studied the influence of the stacking sequences on the low-velocity impact behavior of unidirectional flax fiber reinforced composites and found that cross-ply laminate specimens exhibited the highest peak load and lowest impact energy absorption among all the composite laminates, while quasi-isotropic laminates offered the highest energy absorption and modest peak load values. In addition, Siengchin et al. [21] and Ramakrishnan et al. [22] investigated the impact properties of flax fabric reinforced composites, and both concluded that the incorporation of woven flax fiber textiles could notably improve the impact energy and stiffness of the composites. Bar et al. [23] further compared the low-velocity impact properties of plain-weave flax fabric reinforced composites with those of plain-weave glass fabric reinforced composites and found that the unit mass specific strength and specific energy absorption properties of flax fabric reinforced composites were better than those of glass fabric reinforced composites, which indicated that the flax fabrics had the potential to replace glass fabrics. The aforementioned research clearly shows that the use of flax fiber reinforcement is an effective way to improve the impact properties of composites. However, the impact damage mechanisms of flax fabric reinforced composites, including matrix cracking, fiber breakage, delamination, and adhesive debonding, are extremely complicated and need to be thoroughly investigated.

In order to accurately predict the impact damage mechanism and response of flax fabric reinforced composites, establishing a constitutive model that takes into account the hierarchical structure of composites is a crucial aspect. A damage mechanics-based progressive damage analysis procedure for plain-woven textile composite had been comprehensively introduced in the literature [24], in which the micro-scale and meso-scale failure modes and the ultimate strength of plain-woven textile composite were identified and described. For the modeling analysis of low-velocity impact damage to composite laminates, Bogenfeld et al. [25] reviewed six representative modeling approaches at different scales, ranging from micro-scale to macro-scale. In their work, the advantages and deficiencies of the spring mass model, plate model, layered-shell model, stacked-shell model, and ply-splitting model were introduced in detail. They concluded that a layered-shell model, a stacked-shell model, and a ply-splitting model using cohesive elements to model the interface behavior between each ply could effectively simulate the delamination during the impact loading, and obtain good simulation results. In order to precisely simulate the impact damage of woven fabric reinforced composites, Wu et al. [26] developed yarn-level finite element models of hybrid 3D carbon/glass woven orthogonal composites, including failure criteria and progressive damage behavior. Huang et al. [27] simulated the effect of slits filled with resin on the impact properties of unidirectionally arrayed chopped carbon fiber reinforced composites by using the Johnson-Cook material and failure model to describe the elastic-plastic property of the slits in the finite element models and found that the slits had a negative effect on the load-bearing capacity but increased the energy absorption. The aforementioned research shows that finite element modeling is an effective way to simulate and analyze the damage mechanism of woven fabric reinforced composites under low-velocity impact by introducing accurate failure criteria and progressive damage behavior and building an appropriate geometric model. In most finite element modeling, the yarn of woven fabric reinforcement consists of long synthetic fibers that are twistless and highly parallel to the principal axis direction of the yarn. However, many scholars have found that the yarn twist angle has significant effects on the mechanical properties of the woven flax fabric reinforced composites [28,29]. Therefore, it is necessary to take account of the twist yarn properties in the impact modeling of woven flax fabric reinforced composites.

In this work, plain-woven flax fabric reinforced composite plates were designed and manufactured. Experimental and numerical studies were carried out to investigate the impact response and damage mechanism of the composite plates under different impact energies. Multi-scale finite element models accounting for the twist yarn properties in the software ABAQUS were developed to analyze the damage behavior and failure mechanisms. Numerical results and experimental data were compared to assess the effect of impact energy on the impact response and damage mechanism of the composite plates.

## 2. Experiment

### 2.1. Materials

**Figure 1:**
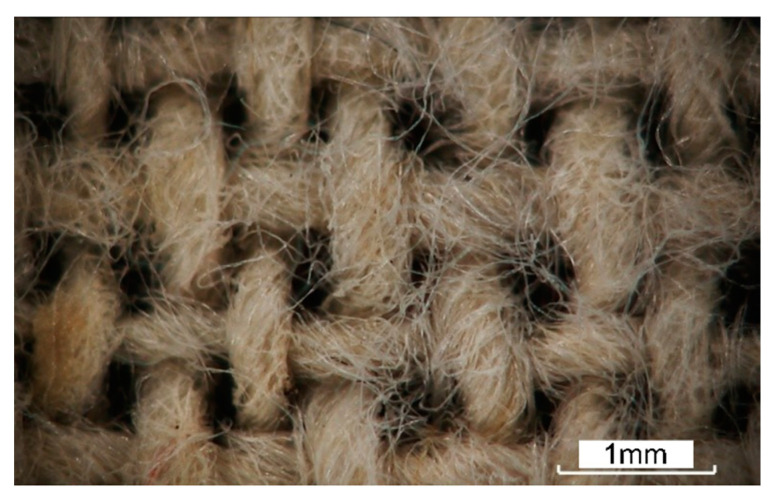
Optical micrograph of plain-weave flax fabric.

Plain-weave flax fabric (the average linear density of flax yarns was 0.085 tex) with an average areal density of 191 g/m^2^ was used as reinforcement, of which the optical micrograph is shown in Figure 1. The polymer resin used as matrix was a thin film of polypropylene, which had a melt temperature of 195 °C. Both the woven flax fabrics and polypropylene films were dried in an oven at 85 °C for 2 h to remove any moisture before use. Furthermore, it can be seen from Figure 1 that the average twist angle of the yarn is about 30°, and the flax fabric has a balanced 2 × 2 twill architecture with the same warp/weft densities of 15 threads/cm.

### 2.2. Composite Fabrication

**Figure 2:**
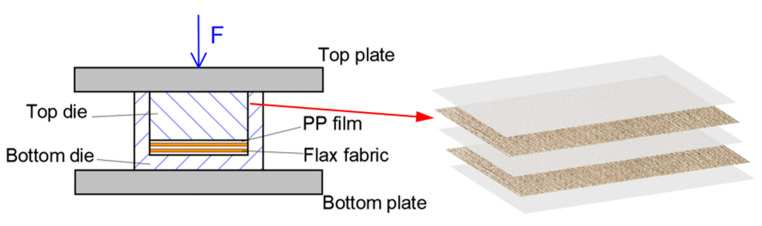
Cross-section of leaky mould.

**Figure 3:**
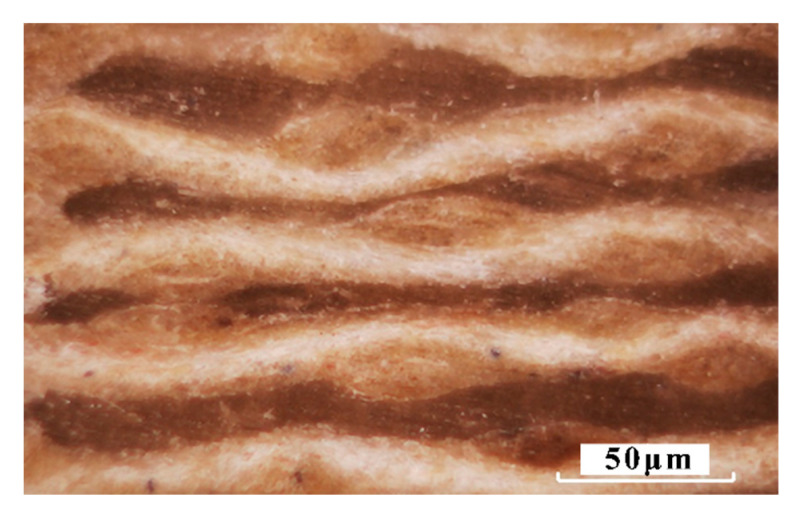
Micrograph of the composite cross-section.

The woven flax fabrics and polypropylene films were cut into and stacked in alternation in a leaky mould, as shown in Figure 2. Both the top and bottom plates of the mould were heated to 210 °C and a constant pressure (10 MPa) was applied to the leaky mould while hot pressing. The total duration of the hot-pressing process was approximately 15 min. The top plate of the press was lifted up to release trapped moisture in the composite every 5 min. Then the leaky mould including the specimen, was moved to a cold press for quick cooling to room temperature under a pressure of 10 MPa. The fiber volume fraction of the final composite plates with seven flax fabric layers was about 34%, and the stacking sequence of flax fabric layers was [0°]_8_. The micrograph of the composite cross-section is presented in Figure 3. The final geometry of the manufactured composites was 120 mm × 120 mm × 3 mm, and the density of the composites was 1.4 g/cm^3^.

### 2.3. Impact Test

**Figure 4:**
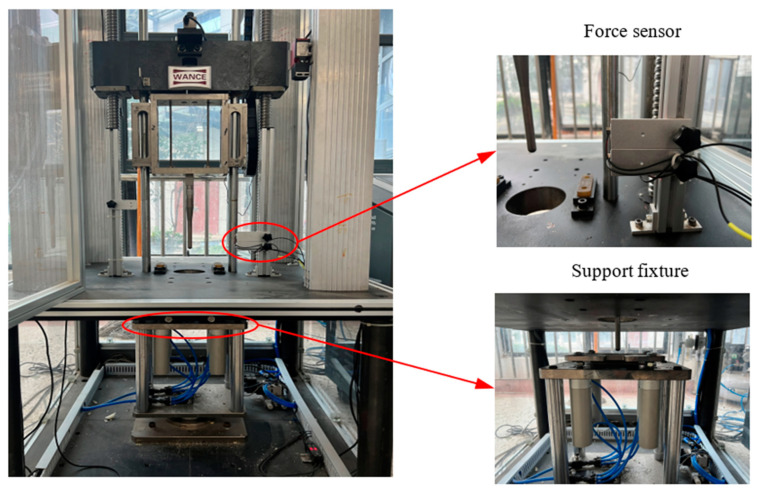
Drop weight testing machine.

In the present study, the low-velocity impact tests were carried out using a drop weight machine based on ASTM D7136, which is shown in Figure 4. As presented in Figure 4, a hemispherical impactor of diameter 12 mm and weighing 4 kg was used in the tests and was equipped with force sensors to measure the contact force between the impactor and specimens. The impacts occurred at the center of the specimens, which were clamped by two fixtures with a central hole with a diameter of 8 cm. As listed in Table 1, the flax fabric reinforced polypropylene composite (FFRPC) specimens were tested at four impact energies (1 J, 2 J, 4 J, and 6 J), referring to the literature [20,22], and are defined as FFRPC-1, FFRPC-2, FFRPC-4 and FFRPC-6, respectively. Each test at the same impact energy was repeated three times. A rebound brake was used to prevent multiple unintentional impacts on the specimens.

### 2.4. Visual Inspection of Specimens

After the impact test, all the specimens were visually checked for any external damage on both the impacted and back surfaces. A digital camera was used to capture scaled images of both the surfaces of the specimens before and after impact.

## 3. Numerical Model

### 3.1. Finite Element Modeling

**Figure 5:**
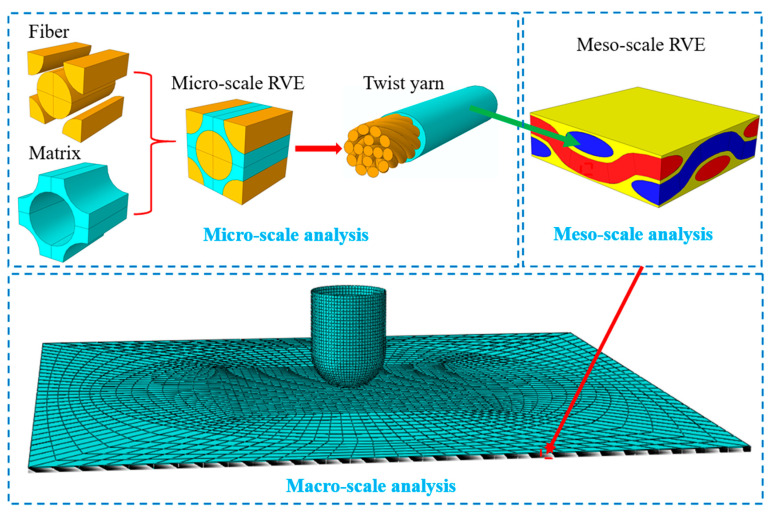
Flowchart of multi-scale analysis.

Two steps were carried out to develop the finite element models of flax fabric reinforced composite plate under low-velocity impact, which is summarized in Figure 5. The first step was micro-scale and meso-scale analysis which predicted the properties of woven flax fabric/polypropylene ply to provide inputs of impact modeling. The second step was macro-scale analysis (Figure 5) which predicted the behavior of the composite plates under impact loading conditions. During both micro-scales, meso-scale and macro-scale analysis, relevant failure criteria were selected to define the failures in the material. All the simulation were conducted using finite element software ABAQUS 6.14, while relevant failure criteria were developed in the material model.

### 3.2. Micro- and Meso-Scale Analysis

**Figure 6:**
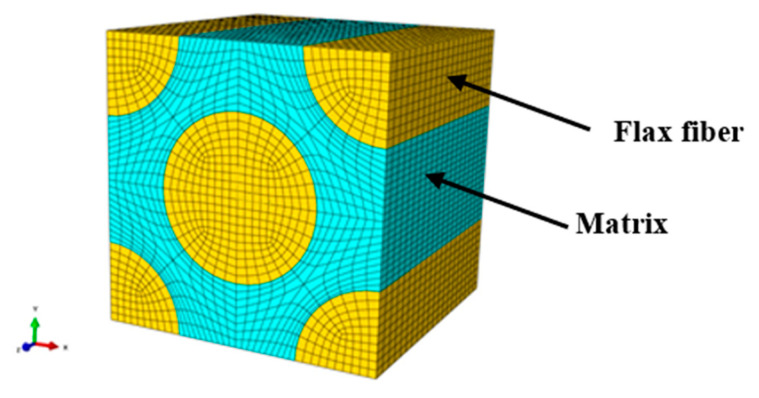
Representative volume element (RVE) in the micro-scale analysis.

According to the micrograph of the composite cross-section (Figure 3) and the meso-scale representative volume element (RVE) of the composite (Figure 5), the yarn volume fraction can be calculated to be about 0.5078. The fiber volume fraction of the final composite plates with seven flax fabric layers was about 0.34, which was determined experimentally by weighing the dry fabric and the resulting composite [28]. Therefore, the fiber volume fraction of the micro-scale RVE consisting of unidirectional flax fibers and polypropylene matrix was around 0.669 (Figure 6), which ignored the effect of twist. In the current finite element analysis, the flax fiber was treated as a transversely isotropic material, while the polypropylene matrix was considered an isotropic material. The mechanical properties of flax fiber and polypropylene are presented in Table 2 and Table 3, respectively. A tie constraint was implemented in the interface between the flax fiber and the matrix to transfer the load. Both flax fiber and matrix were meshed with linear hexahedral elements (C3D8R). In order to calculate the homogenized elastic constants and the strength values of the micro-scale RVE, five loading modes and periodic boundary conditions were applied to the micro-scale RVE, which had been described in detail in our previous work [28]. Furthermore, the maximum stress failure criterion was adopted to describe the damage initiation in the flax fibers, while the von Mises criterion was employed to define the failure in the matrix.

**Table 2:** Mechanical properties of flax fiber [30].

Mechanical Property	Value
Density, *ρ* (g/cm^3^)	1.51
Longitudinal modulus, *E*_11_ (GPa)	54.1
Transverse modulus, *E*_22_ = *E*_33_ (GPa)	7.0
Longitudinal shear modulus, *G*_12_ = *G*_13_ (GPa)	3
Transverse shear modulus, *G*_23_ (GPa)	2
Major Poisson’s ratio, *υ*_12_ = *υ*_13_	0.3
Minor Poisson’s ratio, *υ*_23_	0.75
Longitudinal tension strength, *X*_T_ (MPa)	1000
Longitudinal compression strength, *X*_C_ (MPa)	830

**Table 3:** Mechanical properties of polypropylene matrix [30].

Mechanical Property	Value
Density, *ρ* (g/cm^3^)	1.4
Elastic modulus, *E*_m_ (GPa)	1.6
Poisson’s ratio, *υ*_m_	0.4
Tension strength, *X_m_*_T_ (MPa)	36.1

As shown in Figure 5, the warp and fill yarns of the meso-scale RVE were consisted of twisted flax fibers and polypropylene matrix, while their stiffness matrix can be calculated as follows [28]:

(1) $$f\left(\theta ,\varphi \right)=g\left(\theta \right)g\left(\varphi \right)\;\left(-\theta_{0}\leq \theta \leq \theta_{0},0\leq \varphi \leq 2\pi \right)$$

(2) $$g\left(\theta \right)=\frac{\left|\tan\theta \right|\sec^{2}\theta }{\tan^{2}\theta_{0}}\;\left(-\theta_{0}\leq \theta \leq \theta_{0}\right)$$

(3) $$g\left(\varphi \right)=\frac{1}{2\pi }\;\left(0\leq \varphi \leq 2\pi \right)$$

(4) $$\int_{-\theta_{0}}^{\theta_{0}}\int_{0}^{2\pi }f\left(\theta ,\varphi \right)\;d\theta d\varphi =1$$

(5) $$\left[C^{\prime }\right]=\left[T\right]\left[C^{0}\right]{\left[T\right]}^{T}$$

(6) $$\left[T\right]=\left[\begin{array}{cccccc} l_{1}^{2} & l_{2}^{2} & l_{3}^{2} & 2l_{2}l_{3} & 2l_{3}l_{1} & 2l_{1}l_{2}\\ m_{1}^{2} & m_{2}^{2} & m_{3}^{2} & 2m_{2}m_{3} & 2m_{3}m_{1} & 2m_{1}m_{2}\\ n_{1}^{2} & n_{2}^{2} & n_{3}^{2} & 2n_{2}n_{3} & 2n_{3}n_{1} & 2n_{1}n_{2}\\ m_{1}n_{1} & m_{2}n_{2} & m_{3}n_{3} & m_{2}n_{3}+m_{3}n_{2} & m_{1}n_{3}+m_{3}n_{1} & m_{1}n_{2}+m_{2}n_{1}\\ n_{1}l_{1} & n_{2}l_{2} & n_{3}l_{3} & n_{3}l_{2}+n_{2}l_{3} & n_{3}l_{1}+n_{1}l_{3} & n_{2}l_{1}+n_{1}l_{2}\\ l_{1}m_{1} & l_{2}m_{2} & l_{3}m_{3} & l_{2}m_{3}+l_{3}m_{2} & l_{1}m_{3}+l_{3}m_{1} & l_{1}m_{2}+l_{2}m_{1} \end{array}\right]$$

(7) $$\left[{\bar{C}}_{y}\right]=\int_{0}^{2\pi }\int_{-\theta_{0}}^{\theta_{0}}\left[C^{\prime }\right]f(\theta ,\varphi )d\theta d\varphi$$

where the comprehensive description of above Equations (1)–(7) can be found in our previous work [28].

Whereafter, the mechanical properties of warp and fill yarns obtained on the basis of the simulation results of micro-scale analysis and Equations (1)–(7) were inputted into the finite element models of meso-scale RVE to calculate the homogenized elastic constants and the strength values of the meso-scale RVE using the same five loading modes and periodic boundary conditions as in the literature [28]. In the finite model of meso-scale RVE, the warp and fill yarns were meshed with linear hexahedral elements (C3D8R), as was the matrix meshed with quadratic tetrahedral elements (C3D10). Similarly, the contact between the impregnated yarns and matrix was defined as a “tie constraint”. Finally, the elastic constants and the strength values of the meso-scale RVE with an average twist angle of 30° are listed in Table 4, which would be inputted into the simulation model of macro-scale analysis to predict the behavior of the flax fabric reinforced composite plates under impact loading conditions. As shown in Table 4, the flax fabric reinforced composite is transversely isotropic because of the balanced warp/fill plain weave structure.

### 3.3. Macro-Scale Analysis

**Figure 7:**
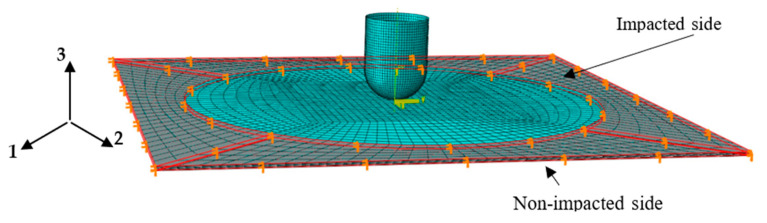
Schematic diagram of boundary conditions of the finite element model for low-velocity impact of composite laminates.

The macro-scale low-velocity impact response model of flax fiber fabric reinforced polypropylene composite laminate was established using the finite element modeling solver ABAQUS/Explicit, as shown in Figure 7. In the impact modeling, the steel impactor was set as a discrete rigid body with a diameter of 12 mm and a height of 16 mm, while it was meshed with the 4-node rigid shell element R3D4. The composite laminate consisted of eight laminated composite plies (intra-laminar) with the same thickness of 0.375 mm and seven interlayer interfaces, while each laminated composite ply was meshed with the 4-node shell element S4R and each interlayer interface between the adjacent plies was meshed with the cohesive element COH3D8. The Hashin damage criteria and the damage evolution response [31] proposed were used, respectively, to predict the damage initiation and evolution in the laminated composite plies, which are presented in Table 5. The quadratic traction-separation law illustrated in Table 6 was used to define the debonding and delamination behavior of the interlayer interface during the impact process, as well as the interface parameters in the simulation, which are listed in Table 7. 

The translational and rotational degrees of freedom of the shell elements in the upper and lower areas clamped by two fixtures were fixed (in Figure 7), while the degrees of freedom in the direction of U1 and U2 of the mass center of the rigid impactor were constrained. Four different initial impact velocities (0.707 m/s, 1 m/s, 1.414 m/s, and 1.732 m/s) were applied to the mass center of the impactor (with a mass of 4 kg) in the U3 direction, corresponding to the initial impact energies (1 J, 2 J, 4 J, and 6 J). The friction coefficient of contact between the impactor and the composite laminate was set to 0.3.

## 4. Results and Discussion

### 4.1. Impact Response

**Figure 8:**
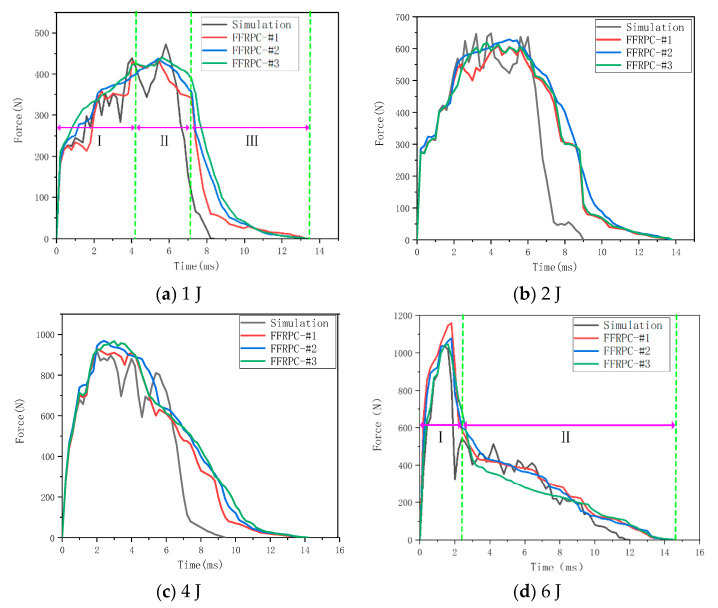
Simulation and experiment results of the force-time response of flax fabric reinforced composites under different impact energies: (**a**) 1 J, (**b**) 2 J, (**c**) 4 J, and (**d**) 6 J.

Figure 8 presents the simulation and experiment results of the force-time response of flax fabric reinforced composites under different impact energies. It can be clearly observed that the peak value of force increases significantly with the increase in impact energy, as does the contact time, which increases slightly. Except for the high impact energy of 6 J, all force-time curves exhibit a similar tendency and can be divided into three stages. In the initial stage (Stage Ⅰ), the impact force increases rapidly while some oscillations occur, presenting relative linearity and indicating little or no damage to the specimen. In Stage Ⅱ, when the impact force rises to a certain level, the curve presents an oscillatory plateau stage with a gradually decreasing impact force trend. That is because the specimen enters the slight damage propagation stage until it reaches the maximum displacement. Finally, the impact force decreases rapidly due to the significant reduction of specimen stiffness with the damage propagation in Stage Ⅲ, while the impactor is rebounded by the specimen [32]. However, for the high impact energy of 6 J, the impact force increases rapidly to the peak value and then quickly declines to a lower value in the first stage due to the serious specimen perforation. In the second stage, the impact force decreases slightly due to the contact friction force caused by the impactor passing through the specimen. Similar results have been reported in the literature [22,26]. Furthermore, it can be seen from Figure 8 that the trends of all impact forces under different impact energies obtained from the finite element simulation show good agreement with those from the impact experiment. The values of peak force show a reasonable match (maximum differences between 3% and 10%).

**Figure 9:**
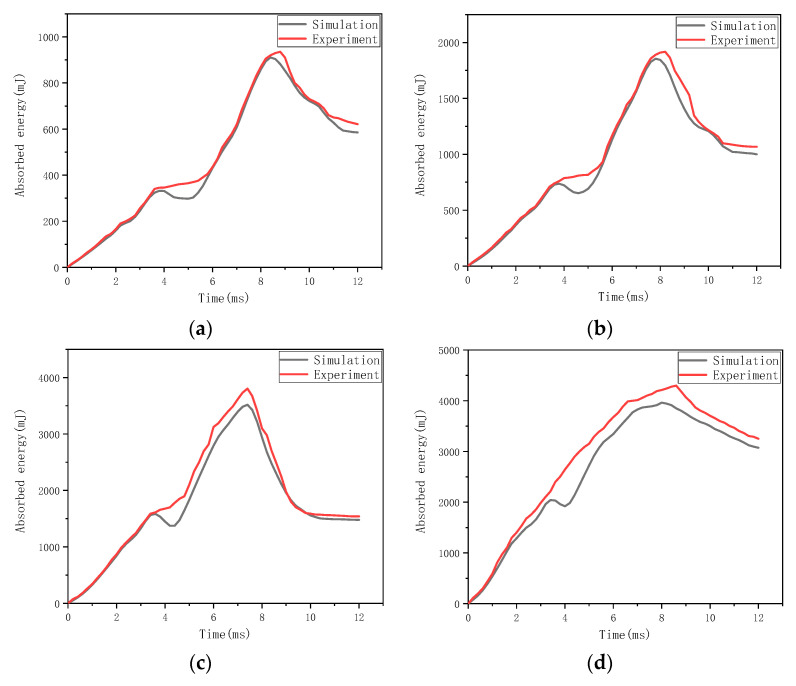
Simulation and experiment results of the absorbed energy-time response of flax fabric reinforced composites under different impact energies: (**a**) 1 J, (**b**) 2 J, (**c**) 4 J, and (**d**) 6 J.

Figure 9 compares the absorbed energy-time response of flax fabric reinforced composites under different impact energies obtained from the simulation results with that from the experimental results. The absorbed energy of the specimen is generally regarded as the sum of the elastic energy and the dissipated energy [26]. As shown in Figure 9, the absorbed energy of all composite specimens increases first during the loading phase of the impact. After the peak is reached, there is a rebound phase where the elastic energy is returned to the impactor. For the specimen under an impact energy of 6 J, the relative returned elastic energy in the rebound phase is much lower than that of other impact energies due to the serious damage to the specimen. There is a good match in the trends of all absorbed energies of the specimen between the results from the finite element analysis and those from the experiment. According to the experimental results, when subjected to impact energies of 1 J, 2 J, 4 J, and 6 J, the maximum values of absorbed energy of specimens are 0.935 J, 1.917 J, 3.85 J, and 4.3 J, respectively, while the corresponding absorption ratio is 93.5%, 95.58%, 96.25%, and 71.7%, respectively. It means that most of the impact energy is absorbed by the specimens when the impact energy is lower than 4 J, and the absorption ratio slightly increases with the increase in impact energy due to the moderate increase in dissipated energy caused by damage such as cracking and fracture. On the contrary, a dramatic decrease occurs in the absorption ratio when the impact energy is 6 J, due to the severe damage to the specimen.

### 4.2. Damage Morphologies

**Figure 10:**
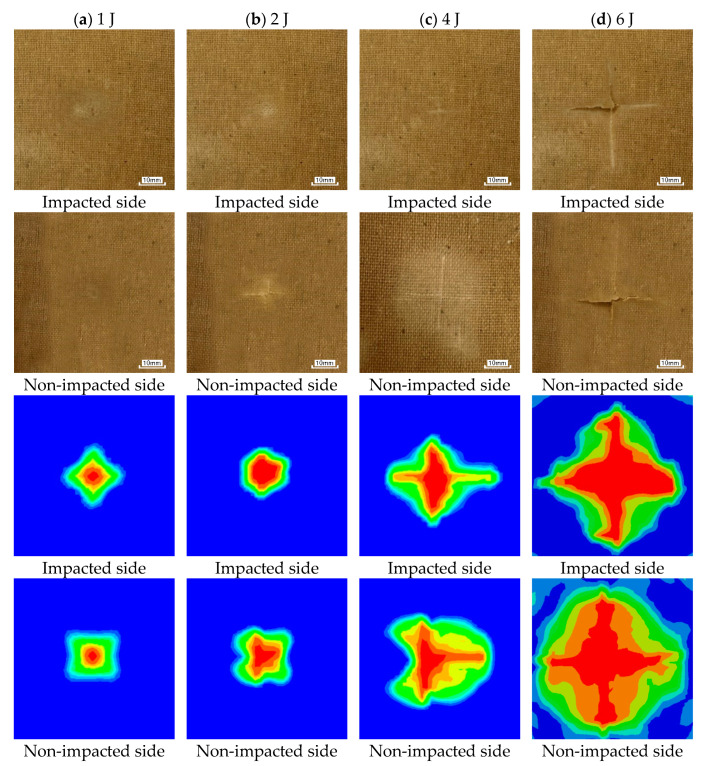
Comparison of the numerical and experimental results of the damage morphologies of both impacted and non-impacted sides for all specimens.

The numerical and experimental results of the damage morphologies of both impacted and non-impacted sides for all specimens under different impact energies are presented in Figure 10. Obviously, the numerical results of the damage morphologies on both sides for all specimens are similar to the experimental results. Under an impact energy of 1 J, only shallow dents occur on both sides of the specimen (Figure 10a). When the impact energy increases to 2 J, a cross-like crack can be observed on the non-impacted side of the specimen, while the impacted side still presents a shallow dent. With the increasing of impact energy, the size of cross-like crack on the non-impacted side increases, as well as the impacted side begins to appear a cross-like crack. The reason causing the phenomenon is a flexural bending failure, in which the fibers and matrix of the non-impacted side suffer from a higher tensile load. Similar results have been published in the work of Ravandi et al. [33]. A dramatic amount of damage can be observed on both sides of the specimen under the impact energy of 6 J. Obvious cracks had also been found on the impact surface of flax fabric reinforced composites at an impact energy of 6 J in the published work of Ramakrishnan et al. [22]. Comparing the numerical and experimental results of the damage morphologies, it can be concluded that the breakage of fibers and matrix appears to extend along the warp and weft directions with increasing impact energy.

In summary, the numerical results of impact force, absorbed energy, and damage morphologies of both sides for all specimens show good agreement with the experimental results, indicating that the established numerical model can effectively predict the impact behavior. Important simulation results will be extracted from the numerical models to further reveal the damage behavior and failure mechanism, which are presented in the following sections.

### 4.3. Impact Resistance and Damage Mechanism

**Figure 11:**
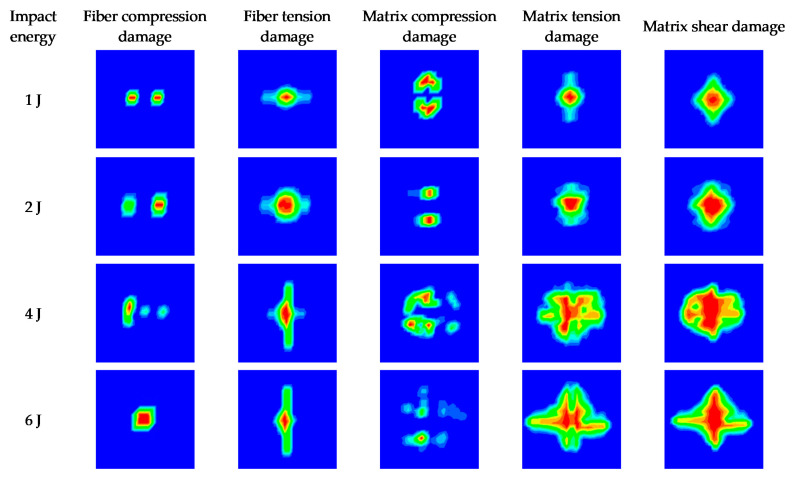
Numerical results of the damage characters of the impacted side for all specimens.

**Figure 12:**
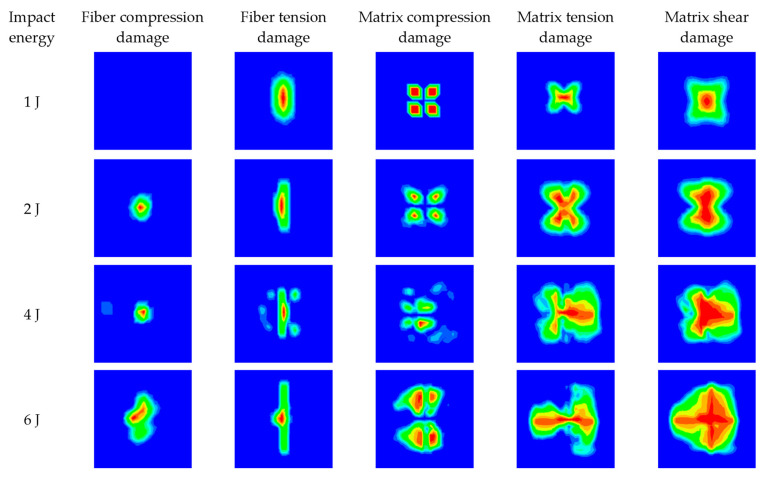
Numerical results of the damage characters of the non-impacted side for all specimens.

Figure 11 and Figure 12 present the damage characteristics of both impacted and non-impacted sides for all specimens under different impact energies. The matrix damage area is larger than that of fiber damage on both impacted and non-impacted sides because the strength of fiber is higher than the matrix. With the increase in impact energy, the matrix tension damage and shear damage on both the impacted and non-impacted sides increase significantly, while the fiber compression damage, fiber tension damage, and matrix compression damage increase slightly. In addition, the simulation results indicate that matrix tension damage and shear damage play a key role in the cross-like crack propagation on both impacted and non-impacted sides with the increase in impact energy. Comparing Figure 11 and Figure 12, it can be found that more fiber tension damage occurs on the non-impacted side, while more fiber compression damage occurs on the impacted side. That is because bending deformation occurs when the composite laminates are subjected to impact loading and contributes to the fiber and matrix on the non-impacted side suffering from more tensile stress.

**Figure 13:**
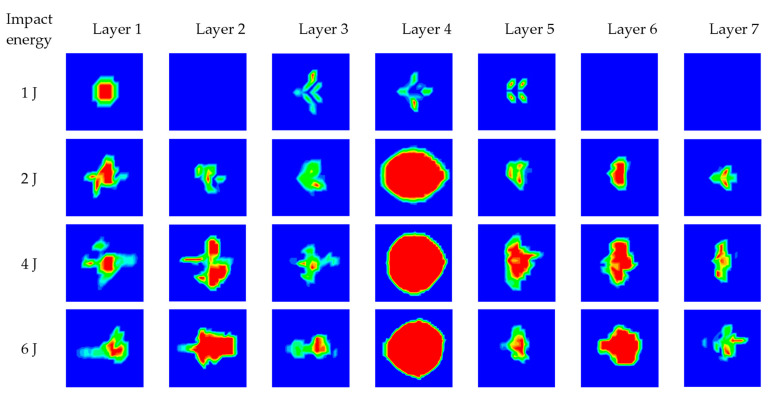
Numerical results of interlaminar damage of the composites under different impact energies.

In order to investigate the delamination resistance of the flax fabric reinforced composite, the interlaminar damage of the composites under different impact energies is presented in Figure 13. The interlaminar damage of the composites is not obvious under a low impact energy of 1 J and dramatically increases when the impact energy increases to 2 J. As the impact energy continues to increase to 4 J and 6 J, the interlaminar damage of the composites increases slightly. Furthermore, it can be clearly observed that the middle layer (layer 4) presents the largest damage area under the impact energy of 2 J, 4 J and 6 J, which shows similar results to the study of Zhao [34]. That is because the bending deformation and the impact stress of the middle layer are also the largest, resulting in relatively serious damage in the region during the impact process. The damage area of each layer shows an approximately symmetric distribution along the middle layer.

## 5. Conclusions

In this paper, a multi-scale finite element analysis strategy is presented to predict the progressive damage of a flax fabric reinforced composite under low-velocity impact. Micro- and meso-scale analyses are conducted to predict the effective properties of the woven unit cell. Macro-scale analysis is carried out subsequently to predict the impact response of composite laminates using the results of micro- and meso-scale analysis as inputs. Based on the studies, the following conclusions are drawn:

- The predicted results of force-time response, absorbed energy-time response, and damage morphologies of both impacted and non-impacted sides using the multi-scale simulation model show good agreement with those of the experiment tests.
- Most of the impact energy is absorbed by the specimens when the impact energy is lower than 4 J, while a dramatic decrease occurs in the absorption ratio when the impact energy is 6 J due to the severe damage to the specimen.
- The simulation results show that matrix tension damage and shear damage play a key role in the cross-like crack propagation on both impacted and non-impacted sides with the increase in impact energy. More fiber tension damage occurs on the non-impacted side and more fiber compression damage occurs on the impacted side because of the bending deformation of the composite laminates subjected to impact loading.
- The interlaminar damage of the composites is not obvious under a low impact energy of 1 J and dramatically increases when the impact energy increases to 2 J. As the impact energy continues to increase to 4 J and 6 J, the interlaminar damage of the composites increase slightly.

## Figures and Tables

**Table 1:** Impact test configuration.

Sample	Impact Energy (J)	Impact Velocity (m/s)	Impact Mass (kg)
FFRPC-1	1	0.707	4
FFRPC-2	2	1	4
FFRPC-4	4	1.414	4
FFRPC-6	6	1.732	4

**Table 4:** Mechanical properties of the meso-scale RVE with a twist angle of 30°.

Mechanical Property	Value
Longitudinal modulus, *E*_11_ (GPa)	7.59
Transverse modulus, *E*_22_ (GPa)	7.59
In-plane Poisson’s ratio, *υ*_12_	0.2
Longitudinal shear modulus, *G*_13_ = *G*_23_ (GPa)	2.748
Transverse shear modulus, *G*_12_ (GPa)	2.2
Longitudinal tension strength, *X*_T_ (MPa)	90
Transverse tension strength, *Y*_T_ (MPa)	90
Longitudinal compression strength, *X*_C_ (MPa)	81.4
Transverse compression strength, *Y*_C_ (MPa)	81.4
Longitudinal shear strength, *S*_13_ = *S*_23_ (MPa)	12.8
Transverse shear modulus, *S*_12_ (MPa)	18.8

**Table 5:** Damage initiation and evolution in the laminated composite plies [31].

	Failure Mode	Initiation Criteria
Damage initiation	$\mathrm{Fiber}\;\mathrm{tension}\;(\sigma_{1}\geq 0)$	$F_{f}^{t}={\left(\frac{\sigma_{1}}{X_{T}}\right)}^{2}+\alpha {\left(\frac{\tau_{12}}{S_{12}}\right)}^{2}$
	$\mathrm{Fiber}\;\mathrm{compression}\;(\sigma_{1}<0)$	$F_{f}^{c}={\left(\frac{\sigma_{1}}{X_{C}}\right)}^{2}$
	$\mathrm{Matrix}\;\mathrm{tension}\;(\sigma_{2}\geq 0)$	$F_{m}^{t}={\left(\frac{\sigma_{2}}{Y_{T}}\right)}^{2}+{\left(\frac{\tau_{12}}{S_{12}}\right)}^{2}$
	$\mathrm{Matrix}\;\mathrm{compression}\;(\sigma_{2}<0)$	$F_{m}^{c}={\left(\frac{\sigma_{2}}{2S_{23}}\right)}^{2}+\left[{\left(\frac{Y_{C}}{2S_{23}}\right)}^{2}-1\right]\frac{\sigma_{2}}{Y_{C}}+{\left(\frac{\tau_{12}}{S_{12}}\right)}^{2}$
Damage evolution	$\sigma =C_{d}\varepsilon$ $d_{I}=\frac{\delta_{I,eq}^{f}\left(\delta_{I,eq}-\delta_{I,eq}^{0}\right)}{\delta_{I,eq}^{f}\left(\delta_{I,eq}^{f}-\delta_{I,eq}^{0}\right)}$ $\delta_{I,eq}^{0}\leq \delta_{I,eq}\leq \delta_{I,eq}^{f}$ $I\in \left\{ft,fc,mt,mc\right\}$ $\delta_{I,eq}^{f}=\frac{2G_{I,c}}{\sigma_{I,eq}^{0}}$	

**Table 6:** Damage initiation and evolution of the interlayer interface between the adjacent plies [31].

Damage Initiation	Damage Evolution
${\left\{\frac{t_{n}}{t_{n}^{0}}\right\}}^{2}+{\left\{\frac{t_{s}}{t_{s}^{0}}\right\}}^{2}+{\left\{\frac{t_{t}}{t_{t}^{0}}\right\}}^{2}=1$	$G_{n}^{C}+\left(G_{s}^{C}-G_{n}^{C}\right)+{\left(\frac{G_{s}+G_{t}}{G_{n}+G_{s}+G_{t}}\right)}^{\eta }=G^{C}$
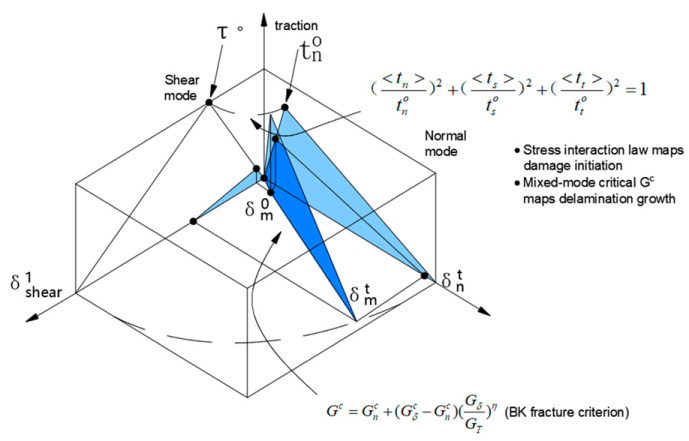	

**Table 7:** Mechanical properties of the interlayer interface between the adjacent plies.

Effective Stiffness (MPa/mm)			Damage Initiation (MPa)			**Fracture Toughness (mJ/mm2)**			**Benzeggagh-Kenane (BK)**
Kn	Ks	Kt	$t_{n}^{0}$	$t_{s}^{0}$	$t_{t}^{0}$	$G_{n}^{C}$	$G_{s}^{C}$	$G_{t}^{C}$	η
16,000	5710	5710	36	20	20	0.8	0.3	0.3	1.75

## Data Availability

Data sharing not applicable.
